# Transcanalicular diode laser-assisted dacryocystorhinostomy

**DOI:** 10.4103/0301-4738.62646

**Published:** 2010

**Authors:** Brigita Drnovšek-Olup, Matej Beltram

**Affiliations:** University Eye Hospital, Medical Centre, Ljubljana, Slovenia

**Keywords:** Dacryocystorhinostomy, lacrimal surgery, minimal invasive surgery, silicone stent intubation, transnasal approach, 980 nm diode laser

## Abstract

**Objective:**

We present a prospective, non-comparative case series study of 126 consecutive diode laser-assisted transcanalicular dacryocystorhinostomy (TCL-DCR) procedures on 122 patients. We analyzed success rate, procedure time and amount of laser energy needed for a 5 mm osteotomy.

**Materials and Methods:**

One hundred and twenty-two patients with nasolacrimal duct obstruction were included in the study. The procedure was performed under general anesthesia, and the nasal mucosa was anesthetized. An endoscope was used for examination of the lacrimal pathways. The site of osteotomy was determined with transillumination of the lateral nasal wall. We achieved osteotomy by applying laser energy via an optic fiber. We used a 980 nm diode laser with power of 10 Watts. We inserted a bicanalicular silicone stent as the last step. Success of procedure was absence of epiphora (subjective), or patency of the lacrimal drainage system on irrigation (objective).

**Results:**

We performed 126 successive endoscopic laser (EL-DCR) with bicanalicular intubation in 122 patients. The average procedure time was 12 min, and on average 245 Joules of laser energy was needed. The silicone stents were removed three to eight months after surgery. We observed absence of epiphora and a patent nasolacrimal duct on irrigation in 105 out of 126 treated eyes. Eighteen patients had epiphora despite a patent nasolacrimal duct on irrigation. This yields a success rate of 83.3%, with an average follow-up period of 12 months.

**Conclusions:**

The 980 nm EL-DCR with bicanalicular intubation is a new contribution to the field of lacrimal surgery. It is a minimally invasive quick procedure yielding a high success rate.

The first intranasal approach was described in 1889 by Killian, and endoscopic dacryocystorhinostomy (DCR) was first performed by Caldwell in 1893, but was soon abandoned due to difficult visualization and numerous complications.[1] However, with the advent of new technology used in endoscopic sinus surgery, permitting better visualization, the endoscopic approach has been revived.[2] This approach permits direct visualization of the lateral nasal wall in the middle nasal meatus, where the osteotomy is performed. The advantages of transnasal endoscopic DCR (TNE-DCR) over external DCR are:[3] No outer skin incision with resulting scar, shorter procedure time, and shorter patient recovery time.

Due to the above mentioned advantages over the classical approach, today TNE-DCR is becoming a treatment of choice for obstructions at the level of the lacrimal sac or nasolacrimal duct and as a revision procedure for failed classical DCR.

The last step in the development of less traumatic DCR is the endocanalicular/transcanalicular approach. In this approach, first described in 1963 by Jack, a probe is inserted through the lower lacrimal punctum via the canaliculus into the lacrimal sac following the anatomical pathway of tear outflow.[4] Osteotomy is performed either by a mechanical drill or laser energy through an optic fiber, which is inserted within the probe.[5]

Lasers with several different wavelengths have been used to perform osteotomy as part of the DCR procedure, mostly as part of a transnasal approach: Holmium:Yttrium-Aluminum-Garnet (Ho:YAG) laser, potassium-tytanyl-phosphate (KTP) laser, Neodymium:YAG (Nd:YAG) laser, Erbium:YAG (Er:YAG) laser, and diode laser. Advantages of laser surgical technique over mechanical DCR techniques include: Precise cutting and removal of tissue by ablation, minimal trauma to adjacent tissue, and mainly, possibility to perform the procedure endoscopically.

The first cadaveric study in 1990 proved that osteotomy of lacrimal bone can be achieved by means of laser energy delivered through an optic fiber by transnasal or endo-canalicular approach.[6] The prefixes endo- or trans- are used as synonyms and mean insertion of an optic fiber inside the lumen of the lacrimal canaliculus and saccus. The authors suggest the term transcanalicular laser DCR, (TCL-DCR), because the endoscope and laser fiber is inserted into the lumen of the canaliculus and used to bypass the obstruction across the lacrimal sac wall (trans-), and not to remove an obstruction inside (endo-) the lacrimal system. The first laser described for clinical use in DCR procedure was the KTP laser in 1993 by Reifler.[7] This was soon followed by the use of Ho:YAG laser and the Nd:YAG laser, as described by Metson *et al*., Piaton *et al*., Dalez and Lemagne, Saint Blancat *et al*., and Woo *et al*. in 1994, 1996 and 1998, notably with an endocanalicular approach in the latter three studies.[8–12] The first descriptions of an endoscopic laser DCR (EL-DCR) with an Er: YAG laser from Emmerich *et al*., and Müllner *et al*. date back to the year 1997 and 1998.[13,14] The use of a diode laser for EL-DCR has been first reported by Eloy *et al*. in 2000, followed by Fernandez *et al*. in 2004.[15,16] Diode laser-assisted DCR is the topic of several current papers, and this laser seems to offer specific advantages for DCR.[17,19]

The main technical obstacles in EL-DCR are to deliver a sufficiently powerful laser beam via a relatively narrow optical fiber, which in turn fits into an endocanalicular probe. Several laser wavelengths successfully comply with this requirement. Yet there are other considerations to take into account, mainly unwanted collateral heating of the probe and residual thermal damage to the target tissue. Based on theoretical and our own preclinical studies, the 980 nm diode laser seems to adequately fulfill all of the above requirements.

The purpose of our prospective study was to analyze the success rate of EL-DCR with a 980 nm diode laser in a series of 126 successive procedures.

## Materials and Methods

Inclusion criteria were: Acquired nasolacrimal duct stenosis, restenosis after TNE-DCR, and congenital nasolacrimal duct stenosis. A complete ophthalmic examination was performed to rule out other causes of watery eyes: Blepharitis, ectropion, entropion, lagophthalmos, trichiasis, conjunctivitis, conjunctivochalasis, keratitis. We performed a Jones dye test and irrigation of the lacrimal pathways. All patients underwent an imaging study (contrast dacryocystorhinography and/or computed tomography) to determine the exact level of obstruction and to evaluate bone thickness. A rhinologic examination was also performed to rule out concomitant nasal pathology, such as septum deviation, concha bullosa, nasal polyposis. Patients with concomitant nasal pathology were referred first to rhinologic treatment. We obtained an informed consent from the patients, and the study has been approved by the national medical ethics committee.

We used a 980 nm diode laser (OptoLight 25, Optotek d.o.o., Ljubljana, Slovenia), in repetitive pulse mode. The laser settings were: Power 10 W, pulse length 90 ms, pause between pulses 50 ms.

**Figure 1:**
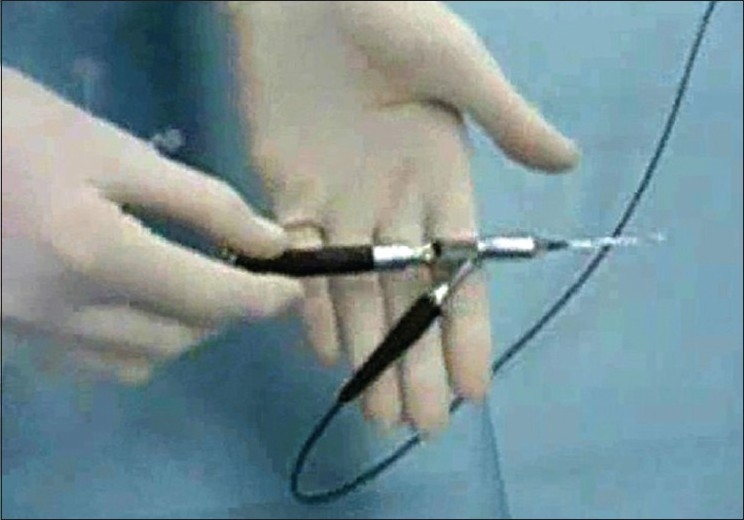
The three-way handpiece with channels for the laser optic fiber, viewing optics and irrigation in assembled state

**Figure 2:**
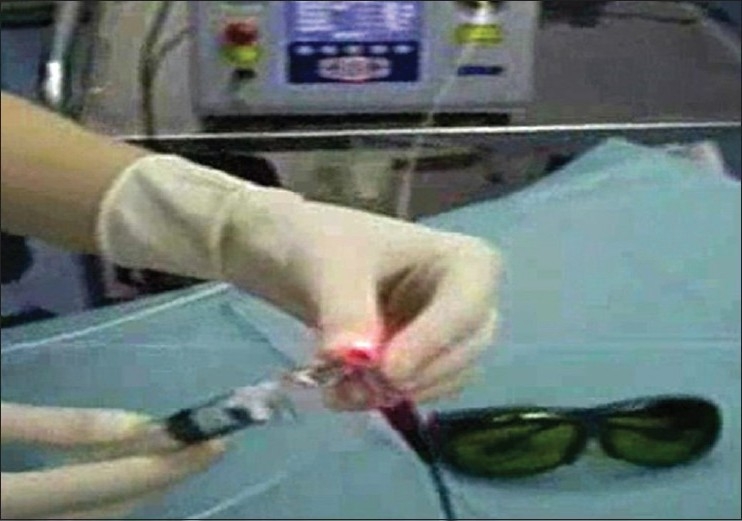
The handpiece is connected to the laser source in the background and syringe with 0.9% saline solution for irrigation. The red aiming beam is visible and pointing to the camera. Safety goggles (bottom right) are mandatory

Laser light was delivered through a 0.5 mm optic fiber, which in turn was inserted into a canalicular probe [Figs. 1 and 2]. The probe contained a second channel for irrigation.

**Figure 3:**
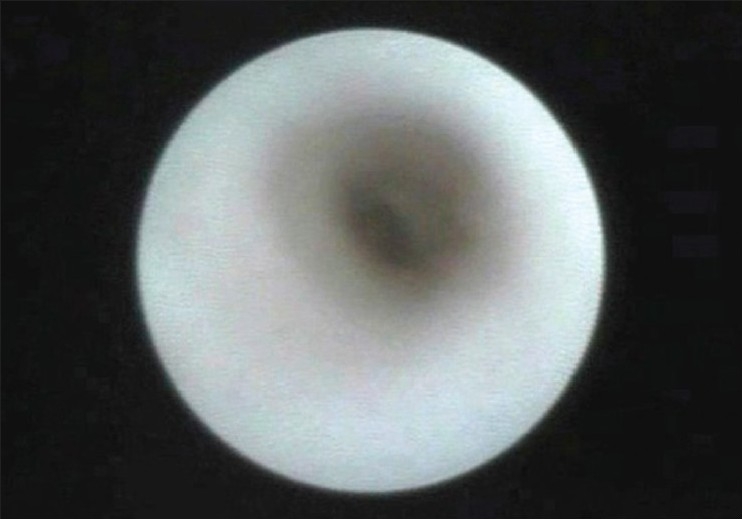
Endoscopic view of a normal lacrimal sac with smooth walls, open lumen containing clear fluid

**Figure 4:**
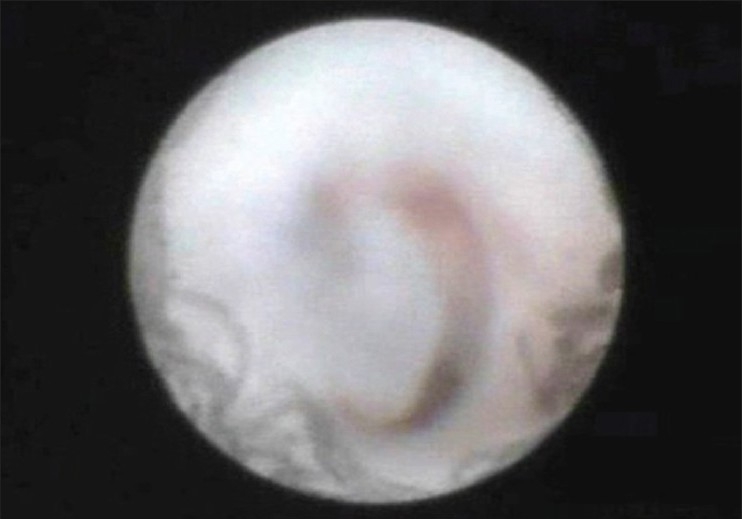
Endoscopic view of a lacrimal sac in nasolcrimal duct stenosis. The lumen is blocked with a mucous plug

**Figure 5:**
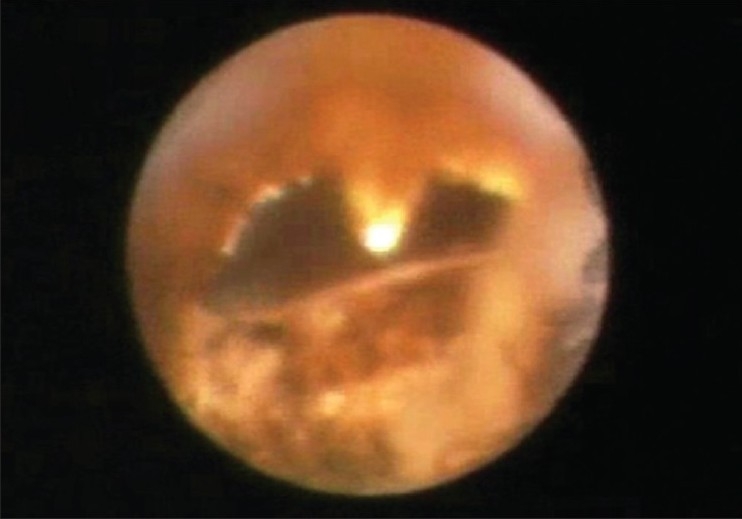
Endoscopic view of the osteotomy (center of the image) achieved with the 980 nm diode laser. No charring of the surrounding tissue can be seen. The tip of the laser fiber is at 12 o'clock. Light from an endonasal endoscope is seen in the middle nasal meatus

The procedure was performed under general anesthesia. The nasal mucosa of the lateral nasal wall was anesthetized by packing with gauze soaked with 5% cocaine solution and infiltration with a solution of epinephrine 1:100.000. An endoscope was inserted through the previously dilated lacrimal punctum and an endoscopic examination of the lacrimal canaliculi and lacrimal sac followed [Figs. 3 and 4]. The site of osteotomy was just anterior and inferior to the attachment of the middle nasal concha determined by transillumination of the lateral nasal wall from the side of the saccus, while inspecting the middle nasal meatus with a nasal endoscope. We performed an infraction of the middle nasal concha in cases of limited view. Osteotomy was achieved by applying laser energy via an optic fiber, which we inserted into the lacrimal sac with a probe [Fig. 5]. Irrigation with 0.9% saline solution of the endocanalicular probe was used to prevent overheating of the probe. The size of osteotomy was controlled through the nasal endoscope. Once an opening of at least 5 mm in diameter has been achieved, application of laser energy ceased.

**Figure 6:**
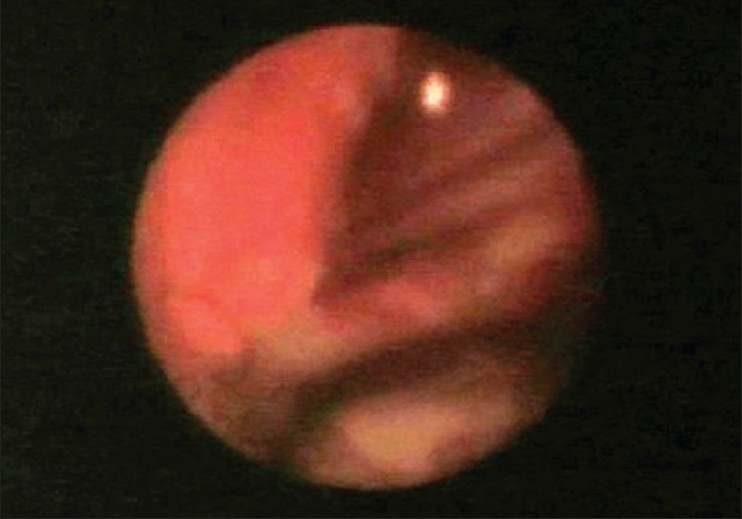
Endoscopic view of the osteotomy from the nasal side. Two silicone tubes were inserted through the osteotomy which is positioned just under the attachment of the middle nasal concha. No charring of the nasal mucosa is seen

The next step in the procedure consisted of intubation with a bicanalicular silicone stent (F.C.I., Paris, France) in the first 44 cases and with two monocanalicular tubes (F.C.I., Paris, France) in the following cases [Fig. 6]. Tamponade of the nasal cavity was performed in cases of bleeding from the mucosa. Postoperative treatment included Dexamethasone-Neomicin-Polymixin B (Maxitrol) eye drops and nasal drops three times daily for three weeks on the treated side as well as 0.9% saline solution six times daily for six weeks applied to the nose on the treated side.

Follow-up examinations were scheduled for the first day, second day, first week postop, three to eight months after surgery and at termination of the study in December 2007. Patency of the nasolacrimal duct was determined as absence of tearing and/or successful irrigation of the lacrimal passages.

We measured procedure time (defined as time from anesthesia of nasal mucosa to fixation of silicone stents), the total amount of laser energy, and carbonization of nasal mucosa.

## Results

We performed 126 successive EL-DCR procedures with bicanalicular intubation in 122 patients between November 2005 and June 2007, four patients underwent bilateral procedures; 89 females and 33 males; (average age 59 years, range 13-84). The average procedure time was 12 min (range 8-35 min). The average total amount of delivered laser energy was 245 J (range 195-685 J), to produce a 5 mm-wide osteotomy. We did not notice any charring or carbonization of the nasal mucosa.

We noticed swelling of the ipsilateral lower eyelid in 89 cases, and bruising in four cases. The swelling usually resolved in a day or two, and bruising was visible for approximately a week.

We removed the silicone stents on average six months after surgery (range three to nine months). In six patients, the silicone stents were removed accidentally while rubbing the eye or cleaning the nose. No other complications were noted.

The patients after silicone stent removal were re-examined at the end of the study (December 2007), the average follow-up time was six months (range 3-12 months). We observed absence of epiphora and a patent nasolacrimal duct on irrigation in 105 out of 126 treated eyes. This yielded a success rate of 83.3%.

## Discussion

The aim of new developments in the field of DCR is to shorten the procedure time, to shorten patient recovery time, to decrease complication rate, to avoid surgical skin and mucosal scars, and to make the procedure possible on an outpatient basis, under local anesthesia.[3] On the other hand, Toti's classic external approach with a 90-95% success rate remains the golden standard, compared to the 80-85% success rate of trasnasal DCR procedures and 70-80% success rate of TCL-DCR.[20,21]

EL-DCR is a minimally invasive surgical procedure. It takes advantage of accessing the operating field through anatomic pathways, in our case this is the lacrimal canaliculus. This contributes greatly to minimizing trauma to surrounding tissue and avoiding unnecessary surgical skin scars. The procedure has a fast learning curve and is in our point of view even easier to learn as compared to classical or TNE-DCR.

Of course there are certain disadvantages of this procedure: Some concern handling of the laser and the costs of it. A second endoscope for endonasal control as well as basic rhinologic surgery training is strongly suggested. We strongly advise all patients with gross anomalies in nasal anatomy to undergo rhinologic treatment first.

When compared to an *ex-vivo* study of the effects of the 980 nm laser beam on chicken breast bone we noticed the following similarities (own unpublished data): There was very limited thermal damage to surrounding tissue, there was no carbonization of bone, and irrigation of the laser probe resulted in limited heating of the probe. On the other hand, we needed less than 100 J of laser energy to produce a 5 mm osteotomy in chicken breastbone as compared to an average of 245 J in EL-DCR.

One of the main open questions is adequate osteotomy size, as restenosis at the site of osteotomy is one of the leading causes of long-term failure in DCR.[22] An osteotomy of more than 10 mm in diameter can be routinely achieved by the classic approach, and a slightly smaller osteotomy of 7-9 mm is achieved with the transnasal approach.[20] The osteotomy size in our series was on an average 5 mm. We believe this is sufficient when using our technique, as there is minimal trauma to the surrounding mucosa and connective tissue, resulting in less postoperative scarring. An interesting computed tomography study by Yazici and Yazici showed that final nasal ostium size six months after surgery is in no correlation with osteotomy size at time of surgery and suturing of mucosal flaps, and measured from 3.1 to 3.8 mm in height.[23] Other factors must play a more important role in the development of restenosis, and we believe this to be tissue trauma with subsequent inflammatory response and scarring.

The patients from our study reported little or no pain postoperatively and were able to leave the hospital the first or second day after surgery, which is a great advantage over classical or TNE-DCR. The amount of lid swelling and bruising was also significantly less compared to classical or TNE-DCR (own observations, unpublished data).

The success rate of transcanalicular diode laser-assisted DCR in our study is among the highest reported for any DCR procedure other than classical DCR.[17,24–26] We explain this by minimal trauma to tissue, which results in minimal postoperative inflammation and scarring.

Additionally, in cases of restenosis, the procedure can be easily repeated (own observations, unpublished data), as there is no scarring of the lacrimal pathways and changement in anatomical relations. Some studies suggest that in future this procedure could be performed even under local anesthesia.[27,28]

## Conclusions

The 980 nm EL-DCR with bicanalicular intubation is a minimally invasive, quick procedure yielding results comparable to classic DCR (90-95%), and better than TNE-DCR (80-85%) or other endoscopic laser DCR procedures (70-80%). It enables fast patient recovery, a short procedure time and avoidance of skin scar.
